# Advancing Interstitial Cystitis/Bladder Pain Syndrome (IC/BPS) Diagnosis: A Comparative Analysis of Machine Learning Methodologies

**DOI:** 10.3390/diagnostics14232734

**Published:** 2024-12-05

**Authors:** Joseph J. Janicki, Bernadette M. M. Zwaans, Sarah N. Bartolone, Elijah P. Ward, Michael B. Chancellor

**Affiliations:** 1 Underactive Bladder Foundation, Pittsburgh, PA 15235, USA; contact@joeja.com; 2 Corewell Health William Beaumont University Hospital, Royal Oak, MI 48073, USA; 3 Department of Urology, Oakland University William Beaumont School of Medicine, Rochester, MI 48309, USA

**Keywords:** urine, biomarker, bladder, interstitial cystitis, inflammation, machine learning

## Abstract

**Background/Objectives.** This study aimed to improve machine learning models for diagnosing interstitial cystitis/bladder pain syndrome (IC/BPS) by comparing classical machine learning methods with newer AutoML approaches, utilizing biomarker data and patient-reported outcomes as features. **Methods.** We applied various machine learning techniques to biomarker data from the previous IP4IC and ICRS studies to predict the presence of IC/BPS, a disorder impacting the urinary bladder. Data were sourced from two nationwide, crowd-sourced collections of urine samples involving 2009 participants. The models utilized included logistic regression, support vector machines, random forests, k-nearest neighbors, and AutoGluon. **Results.** Expanding the dataset for model training and evaluation resulted in improved performance metrics compared to previously published findings. The implementation of AutoML methods yielded enhancements in model accuracy over classical techniques. The top-performing models achieved a receiver-operating characteristic area under the curve (ROC-AUC) of up to 0.96. **Conclusions.** This research demonstrates an improvement in model performance relative to earlier studies, with the top model for binary classification incorporating objective urinary biomarker levels. These advancements represent a significant step toward developing a reliable classification model for the diagnosis of IC/BPS.

## 1. Introduction

Interstitial cystitis/bladder pain syndrome (IC/BPS) is a chronic condition characterized by urinary bladder dysfunction, leading to symptoms such as pelvic pain, increased bladder pressure, urinary urgency, and frequency. Due to the unclear etiology of IC/BPS, no single objective test exists for its diagnosis, making the process complex [1,2,3,4]. Accurate diagnosis typically requires the expertise of a knowledgeable clinician, subjective assessments via cystoscopy, and consideration of patient-reported symptoms. Additionally, bladder wall biopsies may be performed to rule out other confounding disorders, such as overactive bladder, urinary tract infections, and bladder cancer. Bladder biopsy, performed through endoscopy, is a minimally invasive surgical procedure that carries certain risks and is often not preferred by patients.

IC/BPS can be categorized into two main types: non-ulcerative IC (NUIC), which affects the majority of patients and does not present with bladder wall lesions, and ulcerative IC (UIC), characterized by the presence of Hunner’s lesions or diffuse glomerulations. Approximately 10% of individuals with IC exhibit UIC, often presenting with more severe symptoms [5,6,7,8,9,10].

Recent interest has emerged in the utilization of urinary biomarkers and machine learning (ML) techniques for diagnosing and characterizing bladder disorders. For example, a 2019 study [11] comprehensively reviewed the literature for biomarkers used in assessing a variety of conditions with lower urinary tract symptoms. Additional studies examined the measurement of biomarkers related to IC/BPS, such as the levels of IL-6, IL-8, MCP-1, as well as many other markers, to differentiate IC/BPS patients from controls or those with other confusable bladder disorders [12,13,14,15,16,17,18,19].

A study by Jiang et al. [13] with 30 control, 285 NUIC, and 24 UIC subjects utilized statistical modeling applied to ten different biomarkers to distinguish between Control, IC/BPS, and Ulcerative IC/BPS subjects. They found that two of the biomarkers, MIP-1β and TNF-α, provided an ROC-AUC > 0.70 to distinguish IC/BPS from controls, though these had relatively low performance when distinguishing NUIC from UIC (with 50% sensitivity and 39.6% specificity). They also showed that IL-8 and IL-6 are better suited to distinguish NUIC from UIC, whereas MCP-1 is better suited to distinguish between control and IC/BPS. Another study [14] with 30 control, 40 overactive bladder (OAB), and 40 IC/BPS subjects examined the diagnostic utility of urinary biomarkers to differentiate diseased patients with IC/BPS or OAB and controls, as well as between IC/BPS and OAB. MCP-1 and IL-6 were found to distinguish IC/BPS and OAB with ROC-AUC > 0.7.

Our previous work used various biomarkers and other features such as patient reported outcomes (PROs), namely pain scores and the ICSI/PI scores, along with machine learning, to distinguish IC/BPS patients from controls as well as distinguishing the subtypes of IC/BPS [20,21,22]. These studies examined classical supervised and unsupervised machine learning techniques to build models for the purpose of making these distinctions, with an aim to create a more objective diagnostic tool.

In this study, we compiled data from two significant crowdsourcing initiatives, the IP4IC [23,24] and ICRS [20,21,22] studies, which gathered urine samples and associated data from individuals with IC/BPS as well as control subjects. These datasets were combined to create a comprehensive set for training and evaluating machine learning models aimed at classifying the presence of IC/BPS in subjects. This study additionally expands on previous work by adding a new modeling methodology with AutoML.

## 2. Materials and Methods

Crowdsourced Sample Gathering and Analysis. This study utilized data from the IP4IC and ICRS crowdsourcing efforts, which obtained 449 and 1560 samples, respectively, from control, NUIC, and UIC participants. All urine collection cups contained a preservative (Norgen Biotek, Thorold, ON, Canada) that allowed for room temperature storage of samples while stabilizing protein content. In both studies, expression of the urinary cytokines GRO, IL-6, IL-8, and MCP-1 was determined via MILLIPLEX MAP Human Cytokine/Chemokine Multiplex Immunoassay (Millipore Sigma, Burlington, MA, USA) following the manufacturer’s protocol as described previously [22]. Samples were analyzed within three months of arrival at the central testing laboratory. PROs were obtained via participant surveys that collected information such as age, demographics, presence of other diseases and bladder disorders, and bladder symptoms. The Interstitial Cystitis Symptom Index (ICSI, range 0–20 points) and Interstitial Cystitis Problem Index (ICPI, range 0–16 points) were attained by summing results from individual survey questions. Scores with ≥6 points on either index or a combined score ≥ 10 is an indicator of IC [25,26].

Data Preparation. The data used in the modeling were consolidated from the IP4IC and ICRS crowdsourcing efforts. The machine learning features include individual biomarker level measurements (GRO, IL-6, IL-8, MCP-1), as well as the total (summed) ICSI/ICPI scores, as reported by experiment subjects. Both studies provided the same features and used the same methodology for analyte analysis, so these datasets were joined via concatenation.

**Figure 1:**
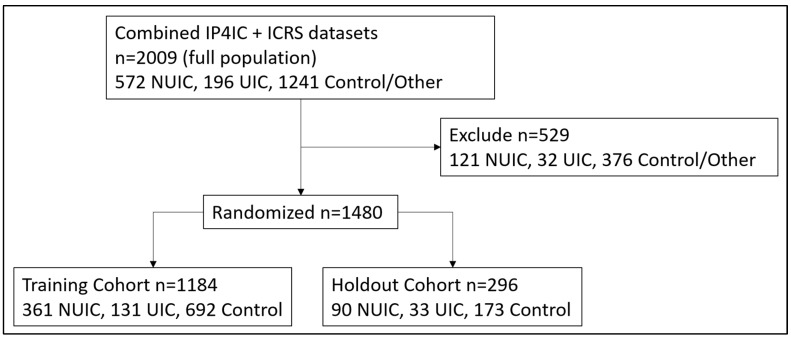
CONSORT Diagram.

The CONSORT diagram in Figure 1 shows the flow of the combined dataset into the training and holdout cohorts for machine learning. Subjects were excluded from modeling if they met the conditions of any of the following categories: (i) subjects with suggested IC presence or symptoms but no formal diagnosis; (ii) prolonged exposure of the urine sample to heat at any point in the collection process; (iii) subjects with active urinary tract infections; (iv) subjects whose samples were collected in a clinic instead of via crowdsourcing; (v) subjects who were positive for SARS-CoV-2 within 6 months of sample collection; and (vi) subjects for which any of the modeling features was not present.

The resulting dataset following exclusions was separated into 80% training and 20% holdout sets via pseudo-random stratified sampling across the three classes, NUIC, UIC, and Control. A random seed value, 1024, was used for reproducibility of this sampling and across the modeling code where applicable (such as with the random forest models).

Experimental Parameters. The machine learning class configuration, feature sets, and models were tested in various permutations.

The classes assigned to a given record were configured both for a three-class classification approach, Control vs. NUIC vs. UIC, and additionally for a binary classification approach, Control vs. IC (any). For the latter approach, classes representing NUIC or UIC subjects were consolidated into a single class.

Three different feature sets were tested: “Biomarkers”, which is the set of the four biomarker levels from GRO, IL-6, IL-8, and MCP-1; “ICSI/ICPI”, which is a single feature that represents the total of both scores; and “Biomarkers + ICSI/ICPI”, which is the combined set of features from the Biomarkers and ICSI/ICPI sets.

The modeling process focused on classification algorithms from two main categories, including classical machine learning methods and an AutoML method. The classical machine learning methods were used in a manner similar to previously published results [20] and include models such as K-nearest neighbors (KNNs), support vector machines (SVMs), logistic regression, and random forest with and without scaling of the feature data. The AutoML package used for the modeling is AutoGluon, version 1.1.1 [27]. The models in the classical ML approach were subjected to varying model hyperparameters. For the AutoML approach, AutoGluon parameters were varied, but the underlying models’ hyperparameters were not explicitly tuned, per suggested AutoGluon operation. A max time limit of six hours was set for each AutoGluon permutation, but in all cases, it was completed before this limit was reached. The sets of hyperparameters tested in both cases are listed in Table 1.

For each permutation of classes, feature sets, and modeling type, the best model was selected via the max F_1_ score (F_1_-macro in the three-class case) based on cross-validation results on the training set. Models are then evaluated according to F_1_, accuracy, and the area under the curve of the receiver-operating characteristic (ROC-AUC) of the holdout set. All permutations were also tested with and without outlier removal using sklearn.ensemble. IsolationForest with contamination = 0.05.

The software to perform the ML modeling was written in Python 3.10.12. The classical ML methods were all provided by the scikit-learn library, version 1.4.0 [28], and the sklearn.model_selection. GridSearchCV class was used to perform the hyperparameter search and model selection with 5-fold cross-validation. The autoML modeling was performed using the autogluon.tablular. TabularPredictor class.

## 3. Results

**Figure 2:**
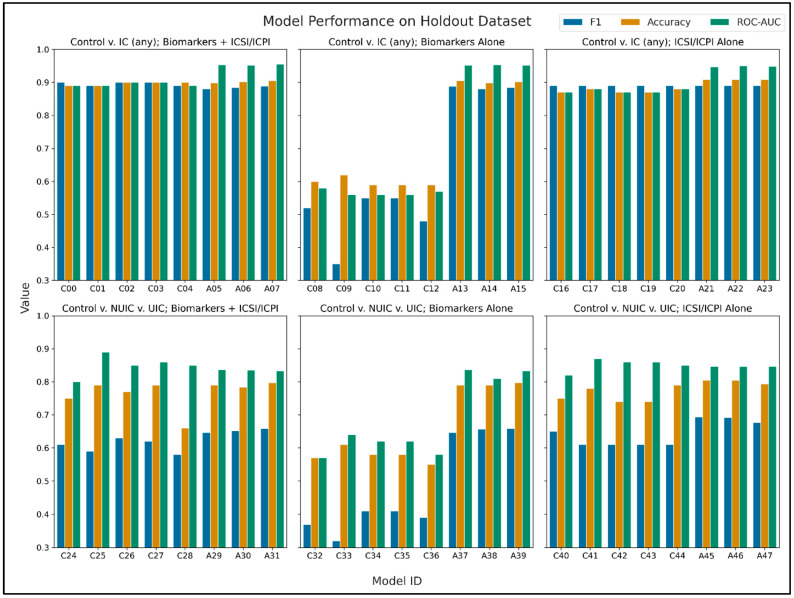
Model performance metrics on the holdout set, F_1_ score, Accuracy, and ROC-AUC score, for the top model from each experimental parameter combination. Model IDs shown here correspond with those reported in Table 1 and Table 2.

**Figure 3:**
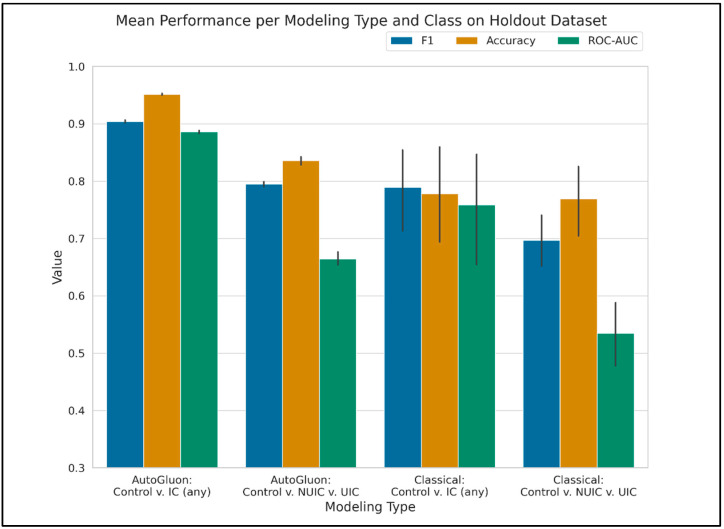
Mean performance at the model type and class level. This was produced by averaging the results over the top models at the modeling type and class configuration level.

**Figure 4:**
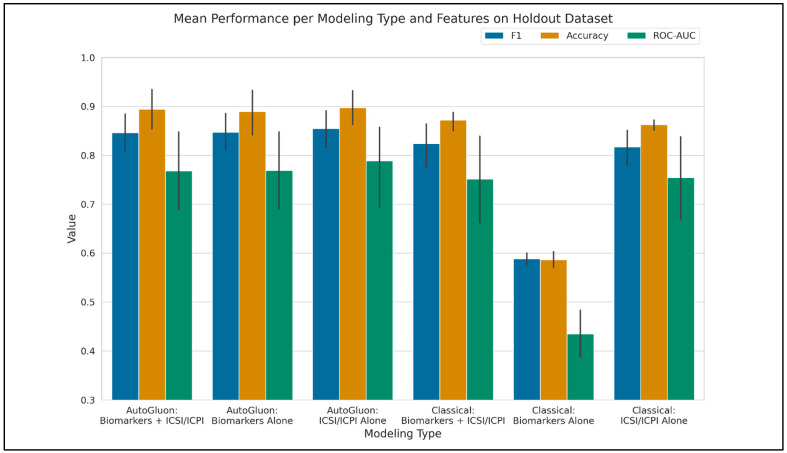
Mean performance at the model type and feature set level. This was produced by averaging the results over the top models at the modeling type and feature set level.

Training and Inference Time. Training time for the AutoGluon permutations was highly variable, with an average of 8073 s, standard deviation of 5554 s, minimum of 2017 s, and maximum of 16,174 s. The classical modeling grid search approach took approximately 1900 s to complete. The models produced by AutoGluon tend to be created as ensembles of many different model types. This leads to an increase in time to make new predictions vs. classical modeling, which tends to produce less-complex models. For example, the average and standard deviation to predict the classes of the holdout set for one of the AutoGluon model permutations using the Biomarkers + ICSI/ICPI feature set, measured with 10 predictions iterations, is 29 ± 2 s. The same set of predictions for one of the random forest model permutations takes 0.01 ± 0.001 s. Modeling performance results on the holdout set are reported in Table 2 and Figure 2, Figure 3 and Figure 4.

Top Model Results. For the binary classification case, when considering the metric used for validation, F_1_, three models tied for the top performance: KNN and random forest with and without scaling (model IDs C00, C02, C03) with F_1_ = 0.90. All of these top performers used the combined Biomarkers + ICSI/ICPI feature set. When considering the maximum ROC-AUC for the binary case, the AutoGluon model A07 had the highest value (F_1_ = 0.89, Accuracy = 0.91, ROC-AUC = 0.96) for the same feature set.

For the three-class case, two AutoGluon models (A45, A46) tied for the top F_1_ score and used the ICSI/ICPI Alone feature set (F_1_ = 0.69, Accuracy = 0.80, ROC-AUC = 0.85 in both cases). When considering the top performance by ROC-AUC, a logistic regression model (C25) scored highest (F_1_ = 0.59, Accuracy = 0.79, ROC-AUC = 0.89) using the Biomarkers + ICSI/ICPI feature set. Confusion matrices for the top models are reported in Table 3.

## 4. Discussion

This work builds upon our previous modeling experiments reported in earlier publications [20,21,22,23,24]. By combining the IP4IC and ICRS datasets for the first time, we achieved improved prediction performance in most instances for both classical and AutoML methodologies when evaluated on the holdout set.

Prior to modeling, we implemented several data exclusions to minimize potential confounding factors. Subjects without a clear diagnosis but exhibiting significant IC/BPS symptoms were excluded to ensure reliable class identification. Samples exposed to excessive heat were also removed to prevent degradation of biomarker levels. Additionally, subjects with urinary tract infections (UTIs) or recent exposure to SARS-CoV-2 were excluded, as the inflammatory cytokines used in this study could be influenced by active infections. We focused exclusively on crowdsourced samples by excluding clinic-collected specimens, allowing for a clearer evaluation of the crowdsourced data. Finally, we omitted subjects with missing data to facilitate modeling without the need for imputation.

In our previous work, the highest ROC-AUC values obtained were 0.87, 0.83, and 0.58 for the feature sets of Biomarkers + ICSI/ICPI, ICSI/ICPI Alone, and Biomarkers Alone, respectively. In this study, we improved the ROC-AUC performance of the combined feature set from 0.87 to 0.96 (a 10% increase, model A07), the ICSI/ICPI Alone feature set from 0.83 to 0.95 (a 14% increase, model A22), and the Biomarkers Alone feature set from 0.58 to 0.95 (a 64% increase, model A14). Notably, AutoGluon models significantly outperformed classical models for the Biomarkers Alone feature set, as illustrated in Figure 2, column 2.

Overall, models distinguishing between control and IC/BPS (any) subjects generally exhibited better performance compared to three-class models. While the feature sets performed reasonably well in the binary classification context, their efficacy diminished when differentiating between control, NUIC, and UIC subjects, resulting in less reliable classifications.

The AutoGluon models demonstrated superior performance over classical models; however, this increased accuracy came at the cost of greater model complexity and higher inference costs, particularly since we utilized the “best quality” preset. Alternative presets are available that may yield faster models, albeit with potentially reduced accuracy.

AutoGluon modeling takes a significantly different approach to modeling versus the classical ML methodology we have presented here and have used previously. The classical approach involves hyperparameter optimization on a single type of model, across different model types. AutoGluon, however, does not perform hyperparameter optimization by default but instead trains on a variety of different model types (many of which were not included in our classical approach), uses bootstrap aggregation, also known as bagging, when training the models, and performs a stack ensembling of the trained models that generally results in a better overall model with desirable predictive capacity [27]. This weighted ensemble of models tends to have increased predictive performance over simpler models. Additional model types that were not included in the classical modeling, such as XGBoost and neural networks, are included in the AutoGluon models, which affords greater flexibility during training and inference. All these benefits are likely contributors to the relatively large increase in F1, accuracy, and ROC-AUC for the “Biomarkers Alone” group over the classical modeling methods, as shown in Figure 4. The differences among other groups were not as significant, likely because the ICSI/ICPI scores provide relatively high predictive capacity alone. Although biomarkers alone yielded the lowest performance among the feature sets, they significantly enhanced classification accuracy when combined with ICSI/ICPI scores, providing a more objective basis for diagnosing IC.

While current results are promising, additional modeling, testing, and validation need to be performed before the models shown here can be considered for clinical diagnosis. The top biomarker + ICSI/ICPI models provide slightly increased performance over the ICSI/ICPI alone models per some metrics. Adding biomarkers to a diagnostic tool aids in increasing the objectivity of IC/BPS diagnosis. The AutoGluon models have provided our best results to date, and this modeling will be expanded in future work. The effect of individual biomarker contributions will also need to be investigated prior to proposing these models as clinical diagnostic tool; all four biomarkers may not be necessary, and the models may benefit by adding measurements of other biomarkers, such as MIP-1β and TNF-α [13]. Additionally, the best future candidate model must be compared exhaustively to current clinical practice to determine the benefit it provides in the diagnosis of IC/BPS.

## 5. Conclusions

In our pursuit of a more objective diagnostic approach for IC/BPS, we successfully developed new models with enhanced classification performance by leveraging datasets from two large crowdsourcing initiatives. This work represents an advancement toward objectivity of IC/BPS diagnosis. An objective laboratory test for diagnosing IC/BPS using an ML model appears both scientifically and clinically viable and useful currently.

## Figures and Tables

**Table 1:** Hyperparameters tested and corresponding model IDs.

Model	Model IDs	Hyperparameters
KNN	C00, C08, C16, C24, C32, C40	{‘n_neighbors’: list(range(1,51))}
Logistic Regression	C01, C09, C17, C25, C33, C41	{‘penalty’: [‘l2’]}
Random Forest	C02, C10, C18, C26, C34, C42	{‘n_estimators’: [50, 100, 200, 500], ‘criterion’: [‘gini’], ‘max_depth’: [None], ‘min_samples_split’: [2, 3], ‘min_samples_leaf’: [1], ‘min_weight_fraction_leaf’: [0.0], ‘max_features’: [None, ‘sqrt’, ‘log2’], ‘bootstrap’: [True, False], ‘class_weight’: [‘balanced’, ‘balanced_subsample’]}
Random Forest (no scaling)	C03, C11, C19, C27, C35, C43	Same as “Random Forest”
SVM	C04, C12, C20, C28, C36, C44	{‘C’: [0.1, 0.5, 1.0], ‘kernel’: [‘linear’, ‘poly’, ‘rbf’, ‘sigmoid’], ‘degree’: [3, 4], ‘gamma’: [‘scale’, ‘auto’], ‘max_iter’: [100,000],}
AutoGluon Configuration 1	A05, A13, A21, A29, A37, A45	num_bag_folds = None, num_bag_sets = None, num_stack_levels = None Fit performed with presets = ‘best_quality’
AutoGluon Configuration 2	A06, A14, A22, A30, A38, A46	num_bag_folds = 5, num_bag_sets = 2, num_stack_levels = 2 Fit performed with presets = ‘best_quality’
AutoGluon Configuration 3	A07, A15, A23, A31, A39, A47	num_bag_folds = 5, num_bag_sets = 3, num_stack_levels = 3 Fit performed with presets = ‘best_quality’

**Table 2:** Results from top model of each permutation of classes, feature set, and model. Model IDs prepended with “C” represent the classical machine learning methods, while those prepended with “A” represent the Results from AutoGluon. As AutoGluon combines different model types, the listed models in this table can vary. Details about the AutoGluon models can be found in Appendix A.

Classes	Feature Set	Model ID	Model	F_1_	Accuracy	ROC-AUC
Control vs. IC (any)	Biomarkers + ICSI/ICPI	C00	KNN	0.90	0.89	0.89
		C01	Logistic Regression	0.89	0.89	0.89
		C02	Random Forest	0.90	0.90	0.90
		C03	Random Forest (no scaling)	0.90	0.90	0.90
		C04	SVM	0.89	0.90	0.89
		A05	WeightedEnsemble_L2	0.88	0.90	0.95
		A06	WeightedEnsemble_L2	0.88	0.90	0.95
		A07	WeightedEnsemble_L3	0.89	0.91	0.96
	Biomarkers Alone	C08	KNN	0.52	0.60	0.58
		C09	Logistic Regression	0.35	0.62	0.56
		C10	Random Forest	0.55	0.59	0.56
		C11	Random Forest (no scaling)	0.55	0.59	0.56
		C12	SVM	0.48	0.59	0.57
		A13	WeightedEnsemble_L2	0.89	0.91	0.95
		A14	WeightedEnsemble_L2	0.88	0.90	0.95
		A15	WeightedEnsemble_L2	0.88	0.90	0.95
	ICSI/ICPI Alone	C16	KNN	0.89	0.87	0.87
		C17	Logistic Regression	0.89	0.88	0.88
		C18	Random Forest	0.89	0.87	0.87
		C19	Random Forest (no scaling)	0.89	0.87	0.87
		C20	SVM	0.89	0.88	0.88
		A21	WeightedEnsemble_L2	0.89	0.91	0.95
		A22	WeightedEnsemble_L2	0.89	0.91	0.95
		A23	WeightedEnsemble_L2	0.89	0.91	0.95
Control vs. NUIC vs. UIC	Biomarkers + ICSI/ICPI	C24	KNN	0.61	0.75	0.80
		C25	Logistic Regression	0.59	0.79	0.89
		C26	Random Forest	0.63	0.77	0.85
		C27	Random Forest (no scaling)	0.62	0.79	0.86
		C28	SVM	0.58	0.66	0.85
		A29	WeightedEnsemble_L3	0.65	0.79	0.84
		A30	WeightedEnsemble_L4	0.65	0.78	0.84
		A31	WeightedEnsemble_L5	0.66	0.80	0.83
	Biomarkers Alone	C32	KNN	0.37	0.57	0.57
		C33	Logistic Regression	0.32	0.61	0.64
		C34	Random Forest	0.41	0.58	0.62
		C35	Random Forest (no scaling)	0.41	0.58	0.62
		C36	SVM	0.39	0.55	0.58
		A37	WeightedEnsemble_L3	0.65	0.79	0.84
		A38	WeightedEnsemble_L4	0.66	0.79	0.81
		A39	WeightedEnsemble_L5	0.66	0.80	0.83
	ICSI/ICPI Alone	C40	KNN	0.65	0.75	0.82
		C41	Logistic Regression	0.61	0.78	0.87
		C42	Random Forest	0.61	0.74	0.86
		C43	Random Forest (no scaling)	0.61	0.74	0.86
		C44	SVM	0.61	0.79	0.85
		A45	WeightedEnsemble_L2	0.69	0.80	0.85
		A46	WeightedEnsemble_L2	0.69	0.80	0.85
		A47	WeightedEnsemble_L2	0.68	0.79	0.85

**Table 3:** Confusion matrices of top models on holdout set. The top model confusion matrices for the binary and three-class case are shown here. Note that for the classical models, the total *N* may be less than 296 (holdout cohort size) due to the use of IsolationForest for outlier removal. AutoGluon models used the full holdout cohort.

			Predicted		
Model ID			Control	IC (Any)	
C00	Actual	Control	133	14	
		IC (any)	15	106	
C02		Control	132	15	
		IC (any)	13	108	
C03		Control	133	14	
		IC (any)	13	108	
A07		Control	156	17	
		IC (any)	11	112	
			Control	NUIC	UIC
C25		Control	133	14	0
		NUIC	10	79	0
		UIC	4	27	1
A45		Control	160	13	0
		NUIC	15	65	10
		UIC	5	15	13
A46		Control	162	11	0
		NUIC	17	63	10
		UIC	5	15	13

## Data Availability

Data are available by request to the authors for non-commercial research use only.

## Appendix A. Trained AutoGluon Models

The following table shows the different model types used to construct each of the top performing models and the number of those models that make up the AutoGluon ensemble. These data were obtained via TabularPredictor.info() on the trained models and accessed via the ‘model_info’ and ‘children_info’ dictionary keys. The number of models in the trained AutoGluon predictors is much higher relative to the classical modeling methodology, which is one reason why there is a greater inference cost with these models.

**Model ID**	**Model**	**Count**
A05	CatBoostModel	10
	GreedyWeightedEnsembleModel	1
	TabularNeuralNetTorchModel	30
A06	GreedyWeightedEnsembleModel	1
	LGBModel	15
	TabularNeuralNetTorchModel	30
A07	CatBoostModel	800
	GreedyWeightedEnsembleModel	1
	KNNModel	2
	LGBModel	704
	NNFastAiTabularModel	984
	RFModel	9
	TabularNeuralNetTorchModel	904
	XGBoostModel	432
	XTModel	9
A13	GreedyWeightedEnsembleModel	1
	TabularNeuralNetTorchModel	80
A14	CatBoostModel	10
	GreedyWeightedEnsembleModel	1
	TabularNeuralNetTorchModel	30
A15	GreedyWeightedEnsembleModel	1
	LGBModel	15
	TabularNeuralNetTorchModel	30
A21	GreedyWeightedEnsembleModel	1
	LGBModel	40
	TabularNeuralNetTorchModel	80
A22	CatBoostModel	10
	GreedyWeightedEnsembleModel	1
	LGBModel	10
	TabularNeuralNetTorchModel	20
	XTModel	1
A23	GreedyWeightedEnsembleModel	1
	TabularNeuralNetTorchModel	15
	XTModel	1
A29	CatBoostModel	800
	GreedyWeightedEnsembleModel	1
	KNNModel	2
	LGBModel	640
	NNFastAiTabularModel	928
	RFModel	9
	TabularNeuralNetTorchModel	848
	XGBoostModel	408
	XTModel	9
A30	CatBoostModel	300
	GreedyWeightedEnsembleModel	1
	KNNModel	2
	LGBModel	240
	NNFastAiTabularModel	365
	RFModel	18
	TabularNeuralNetTorchModel	315
	XGBoostModel	150
	XTModel	18
A31	CatBoostModel	500
	GreedyWeightedEnsembleModel	1
	KNNModel	2
	LGBModel	400
	NNFastAiTabularModel	585
	RFModel	27
	TabularNeuralNetTorchModel	530
	XGBoostModel	250
	XTModel	27
A37	CatBoostModel	800
	GreedyWeightedEnsembleModel	1
	KNNModel	2
	LGBModel	640
	NNFastAiTabularModel	928
	RFModel	9
	TabularNeuralNetTorchModel	848
	XGBoostModel	408
	XTModel	9
A38	CatBoostModel	400
	GreedyWeightedEnsembleModel	1
	KNNModel	2
	LGBModel	320
	NNFastAiTabularModel	460
	RFModel	18
	TabularNeuralNetTorchModel	420
	XGBoostModel	205
	XTModel	18
A39	CatBoostModel	500
	GreedyWeightedEnsembleModel	1
	KNNModel	2
	LGBModel	400
	NNFastAiTabularModel	585
	RFModel	27
	TabularNeuralNetTorchModel	530
	XGBoostModel	250
	XTModel	27
A45	GreedyWeightedEnsembleModel	1
	NNFastAiTabularModel	120
	TabularNeuralNetTorchModel	80
	XGBoostModel	40
A46	GreedyWeightedEnsembleModel	1
	NNFastAiTabularModel	30
	TabularNeuralNetTorchModel	30
	XGBoostModel	10
A47	GreedyWeightedEnsembleModel	1
	NNFastAiTabularModel	30
	TabularNeuralNetTorchModel	30
